# Clinically relevant intronic splicing enhancer mutation in myelin proteolipid protein leads to progressive microglia and astrocyte activation in white and gray matter regions of the brain

**DOI:** 10.1186/1742-2094-10-146

**Published:** 2013-12-05

**Authors:** Adam D Bachstetter, Scott J Webster, Linda J Van Eldik, Franca Cambi

**Affiliations:** 1 Sanders-Brown Center on Aging, University of Kentucky, Lexington, KY 40536, USA; 2 Department of Anatomy and Neurobiology, University of Kentucky, Lexington, KY 40536, USA; 3 Department of Neurology, University of Kentucky, Lexington, KY 40536, USA; 4 Spinal Cord and Brain Injury Research Center, University of Kentucky, Lexington, KY 40536, USA

**Keywords:** Neuroinflammation, Leukodystrophies, Pelizaeus-Merzbacher disease

## Abstract

**Introduction:**

Mutations in proteolipid protein (PLP), the most abundant myelin protein in the CNS, cause the X-linked dysmyelinating leukodystrophies, Pelizaeus-Merzbacher disease (PMD) and spastic paraplegia type 2 (SPG2). Point mutations, deletion, and duplication of the PLP1 gene cause PMD/SPG2 with varying clinical presentation. Deletion of an intronic splicing enhancer (ISEdel) within intron 3 of the PLP1 gene is associated with a mild form of PMD. Clinical and preclinical studies have indicated that mutations in myelin proteins, including PLP, can induce neuroinflammation, but the temporal and spatial onset of the reactive glia response in a clinically relevant mild form of PMD has not been defined.

**Methods:**

A PLP-ISEdel knockin mouse was used to examine the behavioral and neuroinflammatory consequences of a deletion within intron 3 of the PLP gene, at two time points (two and four months old) early in the pathological progression. Mice were characterized functionally using the open field task, elevated plus maze, and nesting behavior. Quantitative neuropathological analysis was for markers of astrocytes (GFAP), microglia (IBA1, CD68, MHCII) and axons (APP). The Aperio ScanScope was used to generate a digital, high magnification photomicrograph of entire brain sections. These digital slides were used to quantify the immunohistochemical staining in ten different brain regions to assess the regional heterogeneity in the reactive astrocyte and microglial response.

**Results:**

The PLP-ISEdel mice exhibited behavioral deficits in the open field and nesting behavior at two months, which did not worsen by four months of age. A marker of axonal injury (APP) increased from two months to four months of age. Striking was the robust reactive astrocyte and microglia response which was also progressive. In the two-month-old mice, the astrocyte and microglia reactivity was most apparent in white matter rich regions of the brain. By four months of age the gliosis had become widespread and included both white as well as gray matter regions of the brain.

**Conclusions:**

Our results indicate, along with other preclinical models of PMD, that an early reactive glia response occurs following mutations in the PLP gene, which may represent a potentially clinically relevant, oligodendrocyte-independent therapeutic target for PMD.

## Background

Microglia are distinguished from other glial cells, such as astrocytes and oligodendrocytes, by the expression of macrophage-associated markers, such as CD11b. Through sampling of the microenvironment, microglia are able to rapidly respond to a disturbance in tissue homeostasis by moving to the potential threat, often in numbers [1,2]. In most diseases of the central nervous system (CNS), an increased expression of macrophage-associated markers, such as major histocompatibility complex (MHC) class II, has been associated with an activation of microglia. Activated microglia in CNS diseases can damage neurons through the production of neurotoxic substances such as tumor necrosis factor-α (TNF-α) [3]. However, the presence of activated microglia in a CNS disease can be a consequence of a tissue disturbance, such as dying cells (neurons or oligodendrocytes), and not a cause of the cell death.

Beyond the responsive, immunological effector like functions, it is now being increasingly recognized that microglia have a number of important physiological functions in the healthy CNS. The housekeeping functions of microglia in the healthy CNS include: 1) phagocytosis of dying cells and cellular debris (such as myelin); 2) synaptic interactions and synaptic pruning; 3) regulation of neuronal activity; 4) suppression of inflammation mediated by inflammatory monocytes; 5) modulating neurogenesis and oligodendrogenesis (for review see: [4-8]). Moreover, genetic mutations affecting microglia function are linked to neurological disease (for review see: [9]); including, both neurodevelopmental disorders (MECP2 in the case of Rett syndrome [10]), and neurodegenerative disorders (CD33 and TREM2 in the case of Alzheimer’s disease [11]) for example.

While still poorly understood, it is believed a heterogeneity exists in microglia depending on the region of the CNS where they are found [8]. The most obvious example would be differences in microglia in white matter versus gray matter regions of the brain. In gray matter, microglia have been shown to be involved in synaptic reorganization [12]. In white matter, microglia facilitate the turnover of myelin [13]. There may also be regional differences in the reactive responses of microglia that make some neuron and glia populations particularly vulnerable to neuroinflammatory responses [14]. Therefore, it is important to understand regional heterogeneity in the microenvironment and in the microglia populations that may influence the reactive glia response and pathology that could result from that response.

Focusing on regional heterogeneity of white matter, recent work has shown that alterations in myelin proteins including proteolipid protein (PLP) and 2′,3′-cyclic-nucleotide 3′-phosphodiesterase (CNP) alone can induce a robust microglia response [15-29]. PLP is the most abundant protein of myelin in the CNS. PLP1 gene encodes for two proteins: PLP, and a smaller splice isoform DM20 that originates from alternative splicing of exon 3B [30]. In the CNS, oligodendrocytes express PLP and DM20 and regulate the expression of these two integral membrane myelin proteins in a developmental fashion. Despite the abundance of PLP in myelin, and the obvious role in neurological disease, PLP1 knockout (KO) mice have only subtle changes in myelin integrity [22,31,32]. However, the PLP1 KO mice do develop a progressive axonal pathology [22].

In humans, mutations in the PLP1 gene cause a spectrum of neurological diseases that vary in severity from the milder spastic paraplegia (SPG2) to the severe connatal Pelizaeus-Merzbacher disease (PMD) [33]. Clinically, deletion of an intronic enhancer (ISEdel) within intron 3 of the PLP1 gene is associated with a mild form of PMD that presents with progressive neurological disability [34]. Recently, a knockin (KI) mouse, PLP-ISEdel, was generated to experimentally define the function of this mutation [35]. The mutation was found to reverse the ratio of PLP to DM20 (less PLP and more DM20), with no change in the total amount of protein from the PLP1 gene. Importantly, the ISEdel mutation in PLP leads to myelin abnormalities that become progressively worse with age [35].

The goal of this study was to determine if the PLP-ISEdel mouse model of a clinically relevant mild form of PMD, that neither lacks nor overexpresses PLP, would be sufficient to induce a reactive microglia response, as seen in more aggressive models of PMD [16,23,24]. In addition, we sought to determine if there was any regional heterogeneity in the microglia response. To this end, the temporal and spatial profile of the reactive glia response (microglia and astrocyte) was measured in the PLP-ISEdel mouse. The reactive glia response was compared to behavioral changes and axonal injury seen in the PLP-ISEdel mouse. We found an early increase in the reactive glia response in the white matter that progressed to involve gray matter regions of the brain. Our results, together with other clinical and preclinical models of PMD, suggest that clinically relevant mutations in PLP1 are associated with a regionally progressive reactive glia response.

## Methods

### Mice

Experiments were conducted in accordance with the principles of animal care and experimentation in the Guide for the Care and Use of Laboratory Animals. The Institutional Animal Care and Use Committee of the University of Kentucky approved the use of animals in this study. The PLP-ISEdel mice were generated as previously described [35]. The PLP-ISEdel mice were backcrossed onto a C57BL/6 J background and maintained as homozygous KI. For controls, aged-matched C57BL/6 J mice were obtained from Jackson Laboratory. All experiments used an approximately 50/50 ratio of female/male mice.

### Open field activity

As previously described [36], mice were placed in an open field maze (50 cm long × 50 cm wide) for 30 minutes, with activity recorded using EthoVision XT 8.0 video tracking software (Noldus Information Technology, Leesburg, VA, USA). The 50 cm × 50 cm open field maze was digitally divided into 25 quadrants of equal size (9 central and 16 peripheral). The nine central quadrants are collectively referred to as the center zone and the 16 peripheral quadrants are collectively referred to as the peripheral zone. Data was scored automatically for distance traveled (cm), velocity (cm/s), and time spent in the center zone versus the peripheral zone.

### Elevated plus maze

As previously described [36], an elevated plus maze (San Diego Instruments, San Diego, CA, USA) consisting of four arms (two enclosed arms and two open arms) elevated 100 cm above the floor was used to assess anxiety-related behavior. The test began with each mouse placed in the center of the maze and the amount of time spent in each arm was recorded automatically by EthoVision XT 8.0 video tracking software (Noldus Information Technology, Leesburg, VA, USA).

### Visible platform water maze

To test the vision of C57BL/6 J and PLP-ISEdel mice, we tested mice on only the visible platform component of the Morris water maze. Briefly, the maze consisted of a common circular swim area of 100 cm. The pool was filled with water until the level was approximately 2 cm below a visible 10 cm circular platform. The platform was placed approximately 15 cm away from the walls in one quadrant of the maze. Mice were given 60 seconds to find the visible platform. After reaching the platform, the mouse was allowed to remain on it for ten seconds and was then removed, dried, and placed in a warming cage until the initiation of that mouse’s next trial after all ten mice in each group had been tested. For each subsequent trial, the mouse was released from a different start quadrant into the maze and allowed to locate the platform. Platform location remained constant throughout testing. Each mouse underwent three trials. Performance was recorded and scored using EthoVision XT 8.0 video tracking software (Noldus Information Technology, Leesburg, VA, USA).

### Nest-building

C57BL/6 J and PLP-ISEdel mice were moved to single house cages in the standard home cage, without a nestlet, at 15:00. A nestlet was added to the cage at 17:00. On the following day at 09:00, two independent observers, blind to the experimental conditions, scored the presence and quality of the nest on a five point scale as follows: 1 = nestlet not noticeably touched, 2 = nestlet partially torn up, 3 = mostly shredded but often no identifiable nest site, 4 = an identifiable but flat nest, and 5 = a (near) perfect nest [37].

### Euthanasia and brain tissue harvesting

Mice were euthanized by sodium pentobarbital overdose, and transcardially perfused with ice-cold PBS for five minutes, followed by ice-cold 4% paraformaldehyde (PFA). The brains were rapidly removed and fixed in 4% paraformaldehyde for 14 to 16 hours, prior to cryoprotection in a 30% sucrose/PBS solution.

### Immunohistochemistry (IHC)

Using a sliding microtome with a freezing stage, serial sagittal sections (30 μm) of one entire hemisphere were collected and the sections were stored in cryoprotectant at −20°C. Staining procedures were conducted on free-floating sections using every twelfth section through the entire hemisphere. Primary and secondary antibodies were diluted in 3% normal goat serum (NGS: LAMPIRE Biological Laboratories, Pipersville, PA, USA, catalog number 7332500) with 0.2% Triton X-100. Endogenous peroxidase activity was quenched with 3% H_2_O_2_ in methanol, prior to the tissue blocking in 10% normal goat serum with 0.2% Triton X-100. Primary antibodies used included: rabbit anti-APP (Life Technologies, Grand Island, NY, USA, catalog number 51–2700; (1:2,000)); rabbit anti-GFAP (Dako, Carpinteria, CA, USA, catalog number Z0334; (1:10,000)); rabbit anti-IBA1 (Wako Chemicals USA, Richmond, VA USA, catalog number 019–19741; (1:10,000)); rat anti-MHCII (I-A/I-E) (BD Biosciences, San Jose, CA, USA, catalog number 556999; (1:5,000)); rat anti-CD68 (AbD Serotec, Raleigh, NC, USA catalog number MCA1957T; (1:5,000)). For the detection of GFAP and IBA1, an HRP conjugated goat anti-rabbit IgG was used. For all other primary antibodies, a biotinylated secondary antibody was amplified in avidin-biotin substrate (ABC kit, Vector Laboratories, Burlingame, CA, USA). All sections were developed in 0.5 mg/ml 3,3-diaminobenzidine tetrahydrochloride solution (Sigma-Aldrich, St. Louis, MO, USA, catalog number D5637). The tissue sections were dehydrated through gradients of ethyl alcohol, and finally xylene. The sections were then coverslipped with Permount Mounting Medium (Fisher Scientific, Pittsburgh, PA, USA).

### Quantitative image analysis of IHC

As previously described [38], the Aperio ScanScope XT digital slidescanner (Aperio, Vista, CA, USA) was used to image the entire stained slide at 20x magnification to create a single high-resolution digital image. The brain regions were outlined using the Aperio ImageScope software (Aperio, Vista, CA, USA). The Aperio positive pixel count algorithm (version 9) was used to quantify the amount of specific staining in the region. The number of positive pixels was normalized to the number of total pixels (positive and negative) to account for variations in the size of the region sampled. Color and intensity thresholds were established to detect the immunostaining as positive pixels and background staining as negative pixels. Once conditions were established for an immunohistochemical stain, the entire batch of slides was analyzed with the same parameters. The resulting color markup of the analysis was confirmed for each slide. Personnel blind to the experimental conditions performed all quantifications.

### Statistics

All statistical analyses were performed using GraphPad Prism Version 6.00, GraphPad software (San Diego, CA, USA). A two-way ANOVA was used for comparisons of age and genotype interactions. Planned comparisons between age-matched C57BL/6 J and PLP-ISEdel mice were made using an un-paired *t*-test, with the exception of the nesting score that used the non-parametric Mann–Whitney *U-*test. Each group used an approximately 50:50 ratio of males to females. Data are expressed as the mean ± the standard error of the mean (SEM). Differences between means from experimental groups were considered significant at the *P* < 0.05 level.

## Results

### Behavior impairments are seen in two-month-old PLP-ISEdel mice

**Figure 1:**
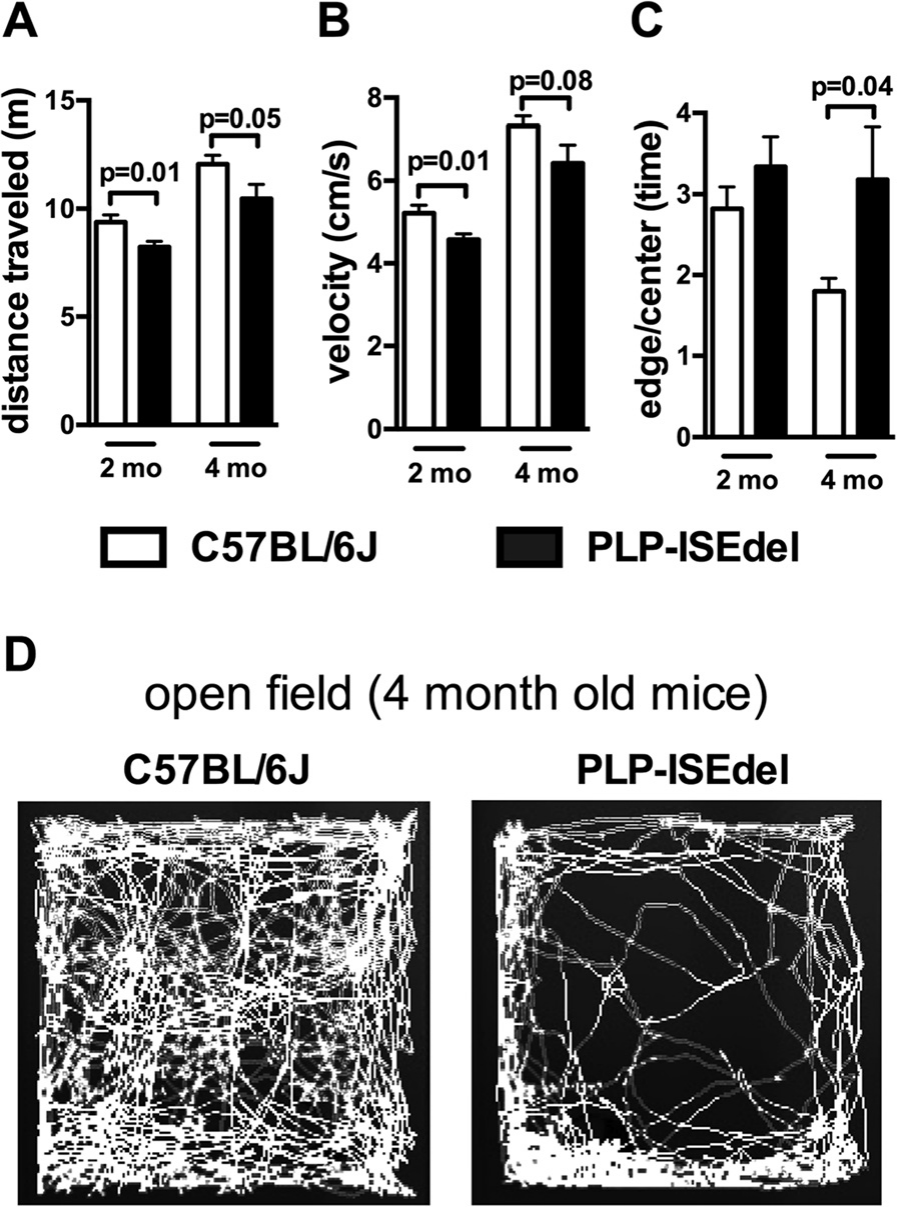
**PLP-ISEdel mice show motor impairments in the open field task.** Mice were placed in an open field maze for 30 minutes and their activity was recorded and analyzed using video tracking software. A significant impairment was found in total distanced traveled **(A)**, and velocity of movement **(B)**. In the 4-month-old group, an age-related difference in thigmotaxis behavior between the wild-type (WT) and PLP-ISEdel mice was found **(C)**, which is shown in a computer-generated trace of the animal’s movements over 30 minutes **(D)**. (2-month-old WT (n = 15) versus PLP (n = 16); 4-month-old WT (n = 12) versus PLP (n = 12).

Clinically, deletion of an ISE within intron 3 of the PLP1 gene is associated with a mild form of PMD that presents with progressive neurological disability [34]. The PLP-ISEdel KI mouse was generated to experimentally define the functional consequences of this mutation [35]. Previously, using the rotorod task, motor deficits of similar severity were found in the PLP-ISEdel mice at 3-months-old and 6-months-old. To extend these findings, the open field behavioral task was used to assess ambulatory movement in two independent groups of 2-month-old and 4-month-old wild-type (WT) and PLP-ISEdel mice. As shown in Figure 1A, total distance traveled over a 30 minute period was decreased in PLP-ISEdel mice compared to age matched WT controls. Total distance traveled was found to increase with age irrespective of genotype. Normalizing the distance traveled by age, such that the 2-month-old mice values were divided by the mean of the 2-month-old WT and the 4-month-old mice values were divided by the mean of the 4-month-old WT, a two-way ANOVA found a significant interaction of genotype (*P* = 0.0016), but no interaction was found for age (as there was no difference between the 2-month-old PLP-ISEdel mice compared to the 4-month-old PLP-ISEdel mice (2-month-old PLP 0.878 ± 0.028; versus 4-month-old PLP 0.867 ± 0.055; mean ± SEM)). Similarly, velocity was found to be decreased in the PLP-ISEdel mice compared to the age matched WT mice (Figure 1B). Again normalizing the velocity by age, a two-way ANOVA found a significant interaction of genotype (*P* = 0.0028), but no interaction for age (2-month-old PLP 0.877 ± 0.027; versus 4-month-old PLP 0.878 ± 0.06; mean ± SEM).

While no progressive deficits were found in the total distance traveled or the velocity of the movement, a progressive change was found in the use of the thigmotaxis behavior in exploring the open field (Figure 1C). Figure 1D shows the video tracking of the movement over the 30-minute period in the open field where 4-month-old PLP-ISEdel mice were found to limit the exploration of the open field primarily to the edges of the maze. As the thigmotaxis behavior could reflect an anxiety-related behavior [39], the 4-month-old WT and PLP-ISEdel group were tested on the elevated plus maze. No significant difference was found in the amount of time spent in the enclosed arms or open arms (closed arms, 4-month-old WT 198.4 ± 13.74; versus 4-month-old PLP 177.3 ± 10.09; open arms, 4-month-old WT 76.08 ± 8.84; versus 4-month-old PLP 77.45 ± 7.87; mean ± SEM(s)). Since we did not find a difference in anxiety-related behavior by the elevated plus maze test, we next sought to determine if the PLP-ISEdel mice had a deficit in visual acuity. Using a visible platform in a water maze to test 4-month-old WT and PLP-ISEdel mice, no significant difference was found in the distance traveled to find the platform (data not shown). The visible platform water maze task suggests that PLP-ISEdel mice are able to use vision to facilitate the escape from the water.

**Figure 2:**
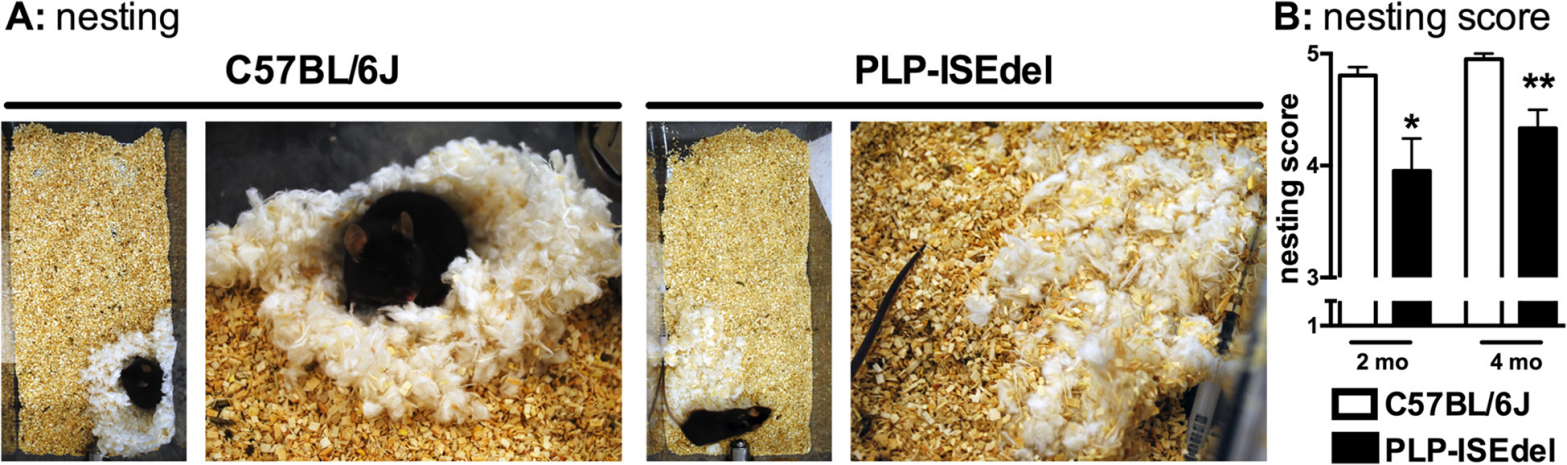
**Nesting behavior is impaired in PLP-ISEdel mice. (A)** Representative images of the types of nests built by the wild-type (WT) mice and the PLP-ISEdel mice. **(B)** At 2-months-old and at 4-months-old, the PLP-ISEdel mice showed a significant impairment in the quality of the nest built compared to the age-matched WT mice. (2-month-old WT (n = 14) versus PLP (n = 16) *P = 0.024; 4-month-old WT (n = 10) versus PLP (n = 6) **P = 0.0039).

Nest-building is a naturalistic mouse behavior, which with the exception of pregnancy and lactation, which is largely related to thermoregulation, but with a significant non-homeostatic component related to exploration and in the wild for camouflage [40,41]. The nest-building behavior was used as a measure of ‘activity of daily living’ to determine if the ISEdel mutation would affect the mouse’s normal behavior performed in the home cages. The 2-month-old and 4-month-old C57BL/6 J mice made a near perfect nest (5 on the nesting scale) as shown in Figure 2A. In comparison, the PLP-ISEdel mice were found to make a lower quality nest that was flat but identifiable (4 on the nesting scale; Figure 2A). Quantification of the nesting behavior by two-way ANOVA found a significant effect of genotype (*P* = 0.0026), but no significant effect of age or interaction of age and genotype.

### Progressive increase in astrocyte activation is seen in the white and gray matter regions of the PLP-ISEdel mice

**Figure 3:**
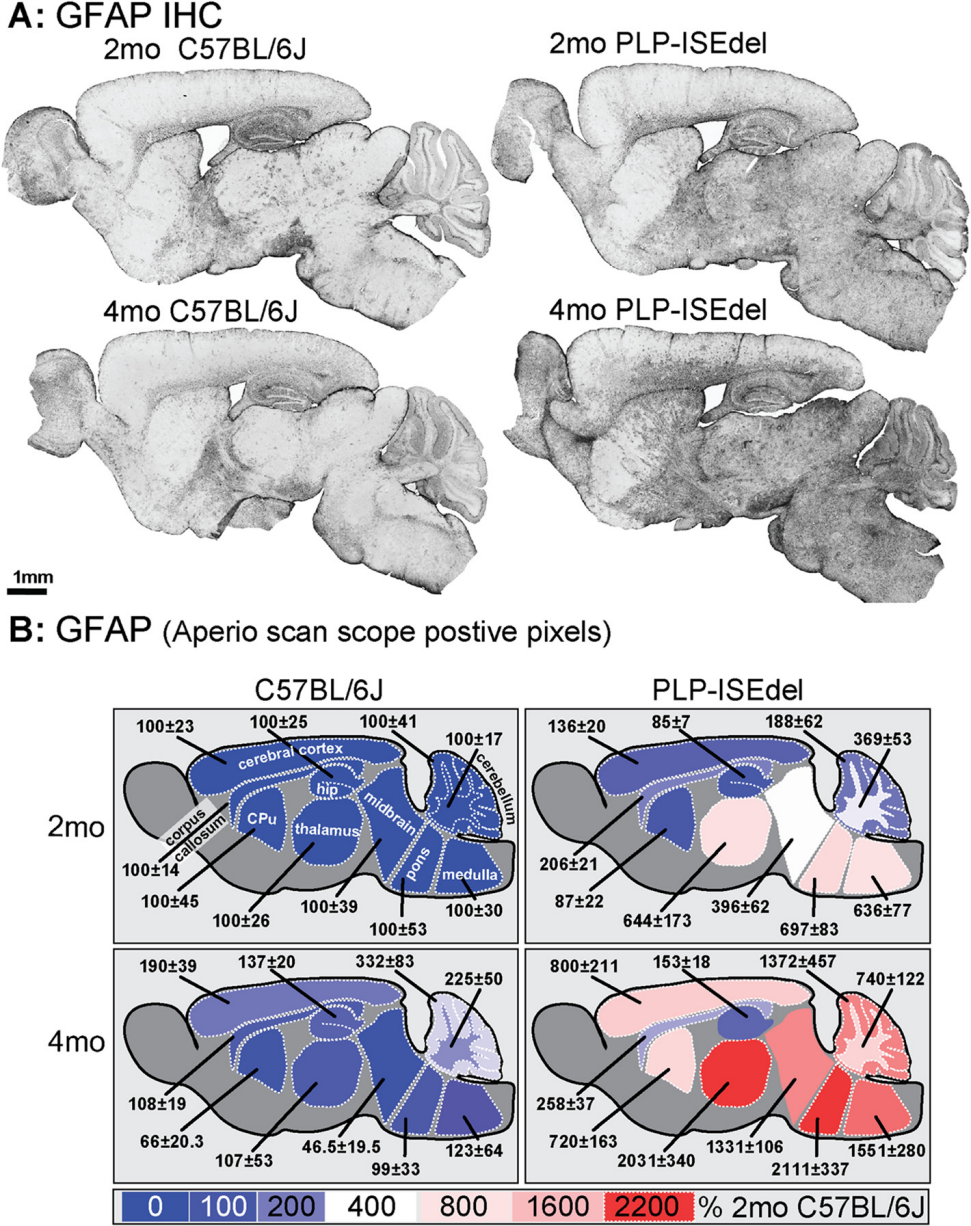
**PLP-ISEdel mice have a progressive astrogliosis throughout the brain. (A)** Strong increase in glial fibrillary acidic protein (GFAP) staining is seen in the PLP-ISEdel mice. **(B)** Quantification of the GFAP staining in the outlined regions is shown as a heat map, with the average of the signal of the 2-month-old wild-type (WT) mice for each brain region used as 100%. Mean ± SEM for each brain region is indicated. (n = 5 to 6 per group). Abbreviations: hippocampus (hip); Cpu, striatum (caudate putamen).

To determine if the PLP-ISEdel mice have a similar reactive glia response as PLP overexpressing mice [16,19,23,24,26-29], we measured specific histological markers of astrocytes and microglia. Glial fibrillary acidic protein (GFAP) is an astrocyte specific marker, which in mice is expressed at low levels in most regions of the brain, but shows a marked increase when the astrocytes become reactive; for example, to injury. With the Aperio ScanScope (Aperio, Vista, CA, USA), using a 20× objective, a single digital image was made of the entire sagittal brain section stained with different glia markers. Figure 3A shows representative images of the regional distribution of the GFAP staining at a low digital magnification. In the WT mice, the majority of GFAP immunohistochemical staining was associated with blood vessels, as well as the typical robust GFAP staining in the hippocampus. In the 2-month-old PLP-ISEdel mice, even at low magnification, a clear increase in GFAP staining was seen in the white matter rich areas of the brain stem and cerebellum. By 4-months-old, the PLP-ISEdel mice showed a strong GFAP staining throughout the brain, including in the cortex.

To quantify the amount of GFAP immunohistochemical staining, regions of interest were outlined corresponding to those shown in Figure 3B. The Aperio ScanScope allows the entire brain structure to be outlined and quantified as a region of interest (ROI), instead of a single microscopic field in a brain region being used as a ROI; thereby, this approach eliminates the potential basis associated with the selection of the ROI. Using the positive pixel algorithm the amount of staining in the regions were quantified (in the 2-month-old and 4-month-old PLP-ISEdel) and expressed as percent of the 2-month-old WT mice for the region (Table 1). As clearly seen in the low power photomicrographs, and supported by the quantitative histopathology, of the ten regions of the CNS included in the analysis, only the hippocampus was spared from an increase in GFAP staining in the 4-month-old PLP-ISEdel mice.

**Table 1:** Summary of quantitative neuropathological analysis of glial fibrillary acidic protein immunohistochemistry (GFAP IHC)

**Brain region**	**C57BL/6 J**		**PLP-ISEdel**		**Two-way ANOVA**		
	**2mo**	**4mo**	**2mo**	**4mo**	**Geno**	**Age**	**Inter**
Cerebellum gm	100 ± 41	332 ± 83	188 ± 62	1,372 ± 457	*	*	*
Cerebellum wm	100 ± 17	225 ± 50	369 ± 53	740 ± 122	****	**	ns
Medulla	100 ± 30	123 ± 64	636 ± 77	1,551 ± 280	****	**	**
Pons	100 ± 53	99 ± 33	697 ± 83	2,111 ± 337	****	***	***
Midbrain	100 ± 39	47 ± 20	396 ± 62	1,331 ± 106	****	****	****
Thalamus	100 ± 26	107 ± 53	644 ± 173	2,031 ± 340	****	**	**
Striatum	100 ± 45	66 ± 23	87 ± 22	720 ± 163	**	**	**
Corpus callosum	100 ± 14	108 ± 19	206 ± 21	258 ± 37	****	ns	ns
Cortex	100 ± 23	190 ± 39	136 ± 20	800 ± 211	**	**	*
Hippocampus	100 ± 25	137 ± 20	87 ±7	153 ± 18	ns	*	ns

2-month-old WT mice used as 100%. Mean ± SEM for each brain region is indicated. (n = 5 to 6 per group). Abbreviations: *P < 0.05, **P < 0.01, ***P < 0.001, ****P < 0.0001; geno = genotype; gm = gray matter; inter = interaction of age and genotype; mo = month-old; ns = not significant; wm = white matter.

### Robust microglia activation is seen in white and gray matter regions of the CNS in the PLP-ISEdel mice

**Figure 4:**
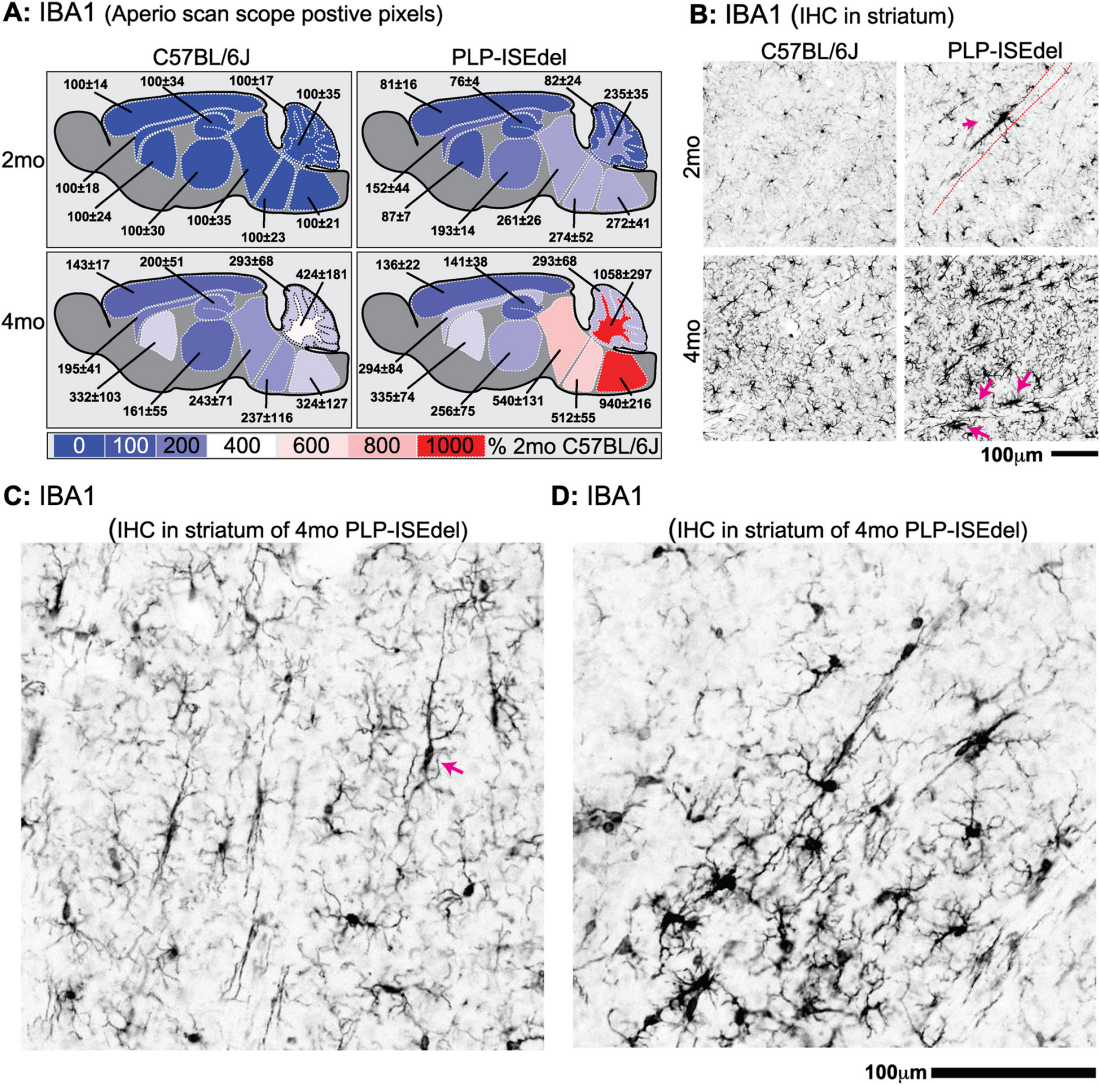
**Ionized calcium-binding adapter molecule 1 (IBA1) staining shows increased microglia activation in PLP-ISEdel mice. (A)** Quantification of the IBA1 staining in the outlined regions is shown as a heat map, with the average of the signal of the 2-month-old wild-type (WT) mice for each brain region used as 100%. Mean ± SEM for each brain region is indicated (n = 5 to 6 per group). **(B)** Morphological changes show microglia activation (magenta arrow) along a white matter tract (indicated by red dashed lines). The microglia exhibited a loss of their normal uniform spatial distribution, and instead showed a more linear distribution (magenta arrow) **(C)** or clusters of strongly activated cells **(D)**.

The ionized calcium-binding adapter molecule 1 (IBA1) is a pan marker of microglia in the brain which is useful for studying morphological changes in the microglia. Activated microglia also upregulate the expression of IBA1; therefore the amount of IBA1 staining can be indicative of microglia activation. As shown by the heat map in Figure 4, quantification of the IBA1 staining showed the strongest activation in white matter rich regions of the brain (Table 2). The amount of IBA1 staining was not significantly increased in the striatum; however, the most striking morphological changes in microglia were seen in the striatum. As shown in Figure 4B, microglia in WT mice were evenly distributed throughout the striatum. In contrast, microglia were found to cluster around white matter tracts in the striatum of the PLP-ISEdel mice. The microglia were also found to have long processes running parallel to the white matter tracts (Figure 4C), and on occasion the microglia would form clusters in areas of the striatum (Figure 4D).

**Table 2:** Summary of quantitative neuropathological analysis of microglia

**Brain region**	**C57BL/6 J**		**PLP-ISEdel**		**Two-way ANOVA**		
	**2mo**	**4mo**	**2mo**	**4mo**	**Geno**	**Age**	**Inter**
**IBA1 IHC**							
Cerebellum gm	100 ± 17	293 ± 68	82 ± 24	293 ± 68	ns	**	ns
Cerebellum wm	100 ± 35	424 ± 181	235 ± 35	1,058 ± 297	*	**	ns
Medulla	100 ± 21	324 ± 127	272 ± 41	940 ± 216	**	**	ns
Pons	100 ± 23	237 ± 116	274 ± 52	512 ± 55	**	*	ns
Midbrain	100 ± 35	243 ± 71	261 ± 26	540 ± 131	**	*	ns
Thalamus	100 ± 30	161 ± 55	193 ± 14	256 ± 75	ns	ns	ns
Striatum	100 ± 24	323 ± 103	87 ± 7	335 ± 74	ns	**	ns
Corpus callosum	100 ± 18	195 ± 41	152 ± 44	294 ± 84	ns	*	ns
Cortex	100 ± 14	143 ± 17	81 ± 16	136 ± 22	ns	*	ns
Hippocampus	100 ± 34	200 ± 51	76 ± 4	141 ± 38	ns	*	ns
**CD68 IHC**							
Cerebellum gm	100 ± 47	500 ± 194	375 ± 88	1,539 ± 398	**	**	ns
Cerebellum wm	100 ± 31	346 ± 67	1,464 ± 127	11,607 ± 214	****	****	***
Medulla	100 ± 11	323 ± 35	1,571 ± 166	7,888 ± 1294	****	****	***
Pons	100 ± 20	433 ± 50	1,161 ± 83	6,121 ± 1107	****	****	***
Midbrain	100 ± 21	260 ± 26	515 ± 76	2,960 ± 379	****	****	****
Thalamus	100 ± 10	251 ± 19	498 ± 78	2,075 ± 257	****	****	****
Striatum	100 ± 29	218 ± 17	157 ± 42	1,142 ± 213	***	****	**
Corpus callosum	100 ± 15	208 ± 22	690 ± 96	2,715 ± 285	****	****	****
Cortex	100 ± 25	427 ± 115	308 ± 62	1,301 ± 285	***	****	**
Hippocampus	100 ± 45	320 ± 24	104 ± 16	513 ± 50	**	****	**
**MHCII IHC**							
Cerebellum gm	100 ± 46	336 ± 63	797 ± 320	632 ± 386	ns	ns	ns
Cerebellum wm	100 ± 75	296 ± 156	493 ± 275	891 ± 223	ns	*	ns
Medulla	100 ± 40	351 ± 57	556 ± 281	1,551 ± 280	*	*	ns
Pons	100 ± 53	437 ± 100	1,587 ± 211	963 ± 291	***	ns	*
Midbrain	100 ± 33	99 ± 22	970 ± 250	1,139 ± 185	****	ns	ns
Thalamus	100 ± 36	109 ± 25	569 ± 200	841 ± 251	**	ns	ns
Striatum	100 ± 25	61 ± 14	326 ± 150	790 ± 318	*	ns	ns
Corpus callosum	100 ± 36	65 ± 19	463 ± 98	2,505 ± 1148	ns	ns	ns
Cortex	100 ± 38	197 ± 54	398 ± 126	474 ± 87	**	ns	ns
Hippocampus	100 ± 47	738 ± 232	1,482 ± 692	1,677 ± 839	ns	ns	ns

2-month-old WT mice used as 100%. Mean ± SEM for each brain region are indicated. (n = 5 to 6 per group). Abbreviations: *P < 0.05, **P < 0.01, ***P < 0.001, ****P < 0.0001; geno = genotype; gm = gray matter; inter = interaction of age and genotype; mo = months-old; ns = not significant; wm = white matter.

CD68 is a macrophage marker, predominantly located in lysosomal membrane, closely related to the family of lysosomal associated mucin-like membrane proteins (lamps) [42,43]. In 2-month-old PLP-ISEdel mice, CD68 staining was localized to white matter rich regions of the brain. By 4-months-old, the CD68 staining was also found in many gray matter rich regions of the brain (Figure 4A,B; Table 2).

**Figure 5:**
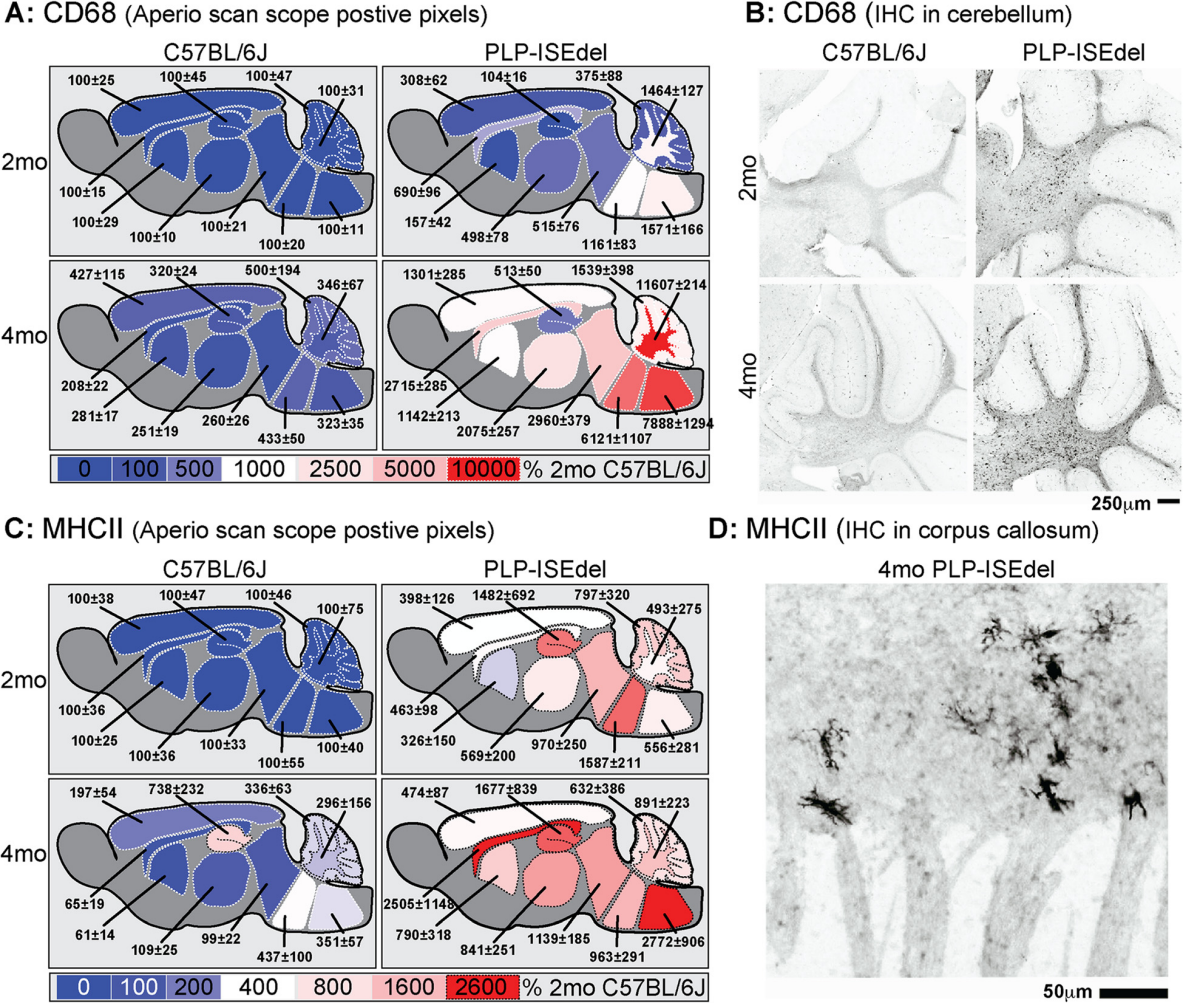
**CD68 and major histocompatibility complex class II (MHCII) histopathology is increased in the PLP-ISEdel mice. (A)** The heat map shows CD68 quantification with 2-month-old wild-type (WT) mice used as 100%. Mean ± SEM for each brain region is indicated (n = 5 to 6 per group). **(B)** CD68^+^ cells were found in the white and gray matter regions of the cerebellum. **(C)** Quantification of the MHCII staining in the outlined regions, expressed as percent of 2-month-old WT as 100%. Mean ± SEM for each brain region is indicated (n = 5 to 6 per group). **(D)** A representative photomicrograph shows MHCII^+^ cells in the corpus callosum of a 4-month-old PLP-ISEdel mouse.

A third marker of microglia, MHCII, was also increased in the 2-month-old and 4-month-old PLP-ISEdel mice compared to the WT control (Figure 5). The increase in MHCII appears to be sensitive to the early changes in microglia activation in the PLP-ISEdel mouse, as widespread changes were seen in the white and gray matter regions of the PLP-ISEdel mouse at both ages (Table 2). However, as shown in Figure 5D, the MHCII staining was sparse which likely contributed to the high variability seen in the PLP-ISEdel mice.

### Four-month-old PLP-ISEdel mice have increased markers of axonal injury

**Figure 6:**
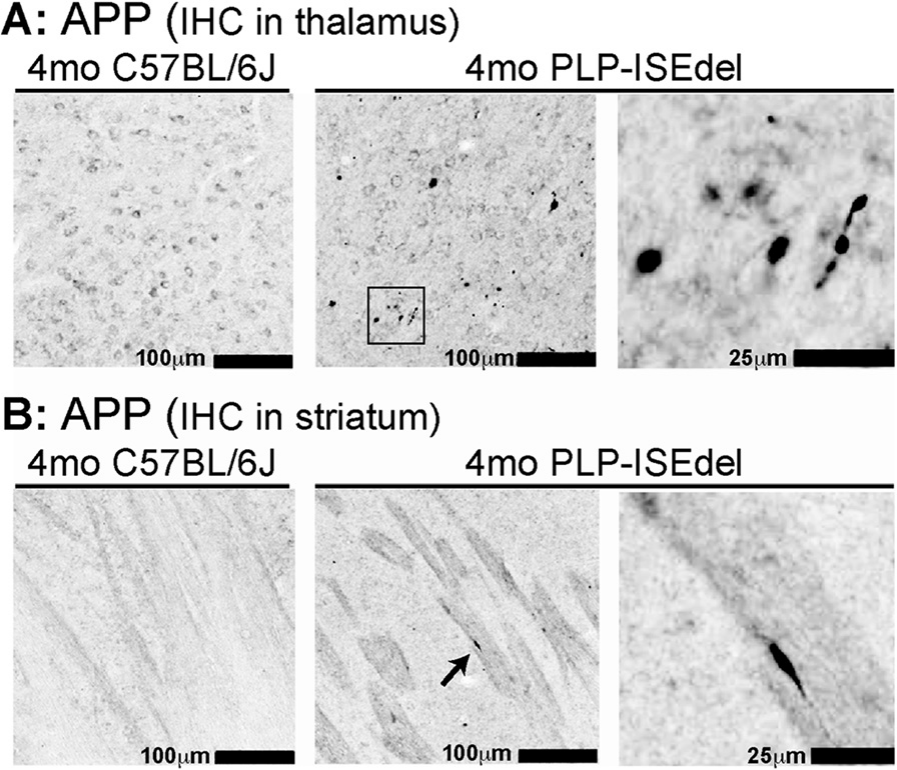
**Amyloid precursor protein** (**APP) accumulation is seen in the 4-month-old PLP-ISEdel mice. (A)** In 4-month-old wild-type (WT) mice, APP staining was localized to the neuronal cell body and no APP^+^ spheroids were found. In 4-month-old PLP-ISEdel mice, many APP^+^ spheroids were seen in the thalamus. The black box shows the region of the higher power image on the right. **(B)** In the striatum, APP accumulation was seen in the white matter tracks in the 4-month-old PLP-ISEdel mice but not in the WT mice. Arrow indicates the APP^+^ spheroid shown in the higher power image on the right.

Previously, we reported that axonal degeneration was not seen in the optic nerve of the PLP-ISEdel mouse up to six months of age [35]; however, other regions of the brain were not investigated. Using amyloid precursor protein (APP) immunohistochemistry as a marker for axonal injury, we sought to determine if axonal accumulation of APP could be seen in other regions of CNS in the PLP-ISEdel mice. In 2-month-old WT and 2-month-old PLP-ISEdel mice, axonal accumulation of APP was not detected. However, in all of the 4-month-old PLP-ISEdel mice included in the study, APP^+^ spheroids were found while APP^+^ spheroids were not detected in the 4-month-old WT mice. Some APP^+^ spheroids were seen in white matter rich regions of the corpus callosum and brainstem of the 4-month-old PLP-ISEdel mice. However, the most robust APP^+^ spheroids were found in the thalamus (Figure 6A) followed by the striatum (Figure 6B).

## Discussion

PLP is the major myelin protein of the CNS. Point mutations and deletions/duplications of the PLP1 gene are associated with X-linked dysmyelinating leukodystrophies, PMD and SPG2 [33]. Duplication of the normal PLP1 gene is associated with approximately 70% of PMD cases [44,45]. Point mutations in the PLP1 gene are a minority of the PMD and SPG2 cases, but can cause the entire spectrum of the PMD and SPG2 clinical manifestations [46]. Recently, we have identified in a group of PMD patients that a deletion in the PLP1 intron 3 splicing enhancer caused a mild form of PMD [34]. The PLP-ISEdel mouse was generated to investigate the mechanism by which loss of the intron 3 splicing enhancer leads to the progressive neurological disability, demyelination, and axonal loss in the PMD patients with this PLP1 gene mutation [35]. As we previously reported, by electron microscopy analysis of the optic nerve, myelin in the PLP-ISEdel mouse forms normally although there are redundant loops of myelin at one month. At three and six months, myelin compaction is progressively abnormal suggesting myelin instability; however, changes in the levels of myelin basic protein and CNP in the optic nerve were not found [35]. We report here the temporal and spatial profile of microglia and astrocyte activation in the PLP-ISEdel mouse and the regional heterogeneity of the glial activation response. We also compared the temporal onset of the glia activation to behavioral changes and to a marker of axonal injury (APP). We found that motor behavior abnormalities were present at the earliest age investigated (two-months-old). In addition we found a reactive glia response at 2-months-old in the PLP-ISEdel mice. While the motor impairments did not worsen in the 4-month-old group of PLP-ISEdel mice, we did find a widespread increase in markers of a reactive glia response with age. In addition, in the 4-month-old PLP-ISEdel mice, a marker of axonal injury was increased.

The motor deficits in the open field test and the impairment in nesting behavior occurred in the PLP-ISEdel mice at two months of age. This is in agreement with our previous report, which found motor impairments using the accelerating rotarod at three months of age in the PLP-ISEdel mice [35]. The motor impairments in the PLP-ISEdel mice were not progressive between two months of age and four months of age, supporting our previous work using the rotarod [35]; however, increasing motor impairments at later stages cannot be ruled out. This is in contrast to progressive motor impairment seen in the PLP-null mouse; however, these motor impairments are not evident until after the mice are 16 months of age [22]. In comparison, the recently generated PLP1dup mice, which have a single duplication of the PLP1 gene as found in the majority of PMD patients, have a progressive motor impairment beginning at approximately four months of age [21]. We found a progressive, age-related change in the search strategy used to explore the open field, as the 4-month-old PLP-ISEdel mice demonstrated increased use of a thigmotaxis behavior. While the thigmotaxis behavior did not appear to reflect anxiety or visual deficits, the possibility that visual deficits influenced the thigmotaxis behavior should not be excluded.

A major finding in this study is the widespread early and progressive astrogliosis in white and gray matter regions of the PLP-ISEdel mice. In the 2-month-old mice, the astrogliosis began in the cerebellum, brain stem and thalamus. By 4-months-old, the PLP-ISEdel mice showed increased astrogliosis throughout the brain. Neuropathological assessments of PMD patients with PLP1 duplication, deletion, and mutations are in agreement with our findings. In the reported cases with mutations in PLP1, astrogliosis was localized to the cerebellum. In the patient cases with PLP1 duplication, strong astrogliosis was found in the striatum, thalamus, cerebellum, and cerebral cortex. It should be noted that the cases reported with mutations in PLP1 were significantly younger at time of death (approximately 20-years-old) than the majority of the reported cases with PLP1 gene duplication (approximately 50-years-old). In one younger case with PLP1 gene duplication (37 years), astrogliosis was less pronounced [47]. Therefore, the astrogliosis in the PLP-ISEdel mice appears to follow a clinically relevant disease progression.

Our findings of robust microglia activation in the CNS in both white and gray matter regions of the PLP-ISEdel mice add to the growing body of literature which suggests that disruptions in PLP protein promote microglia activation [15-24] (see Table 3). In agreement with our findings, a similar pattern of microglia activation to the PLP-ISEdel mice has been reported in PLP1 transgenic mouse (PLP1tg) [16,19]. Specifically, the number of IBA1^+^ microglia was significantly increased in the cortex and striatum of 1-month-old PLP1tg mice. In the striatum of the PLP1tg mice, microglia processes were found to follow axonal paths, as we report here for the PLP-ISEdel mice. A progressive age-related increase in the number of IBA1^+^ and CD11b^+^ cells was also reported for PLP1tg mice [16,19], supporting the findings of the current study (Table 3).

**Table 3:** Proteolipid protein (PLP) mutant mouse models show glial activation

**Mouse model**	**Type of mutation**	**PMD**	**Major pathology**	**Reactive glia response**
PLP1-null [26]	Targeted KO of PLP and DM20	yes [48,49]	Myelin ↔ / OL # ↔ / motor ↓↓↓ @ 16mo / normal lifespan	Astrocytes ↑↑ / microglia ↑↑ after 12 months [22]
PLP-ISEdel [35]	KI of intronic splicing enhancer	yes [34]	Myelin ↔ / OL # ↔ / motor ↓ / normal lifespan	Astrocytes ↑↑↑ / microglia ↑↑↑ [current]
PLP1dub [21]	Genomic duplication PLP1 locus	yes [50]	Myelin ↓ / OL # (nr) / motor ↓↓ / lifespan (nr)	Astrocytes ↑↑ / microglia ↑↑ [21]
PLP1tg [20]	Overexpresses native PLP1		Myelin ↓↓ / OL # ↓↓ / motor ↓↓↓/ lethal (2 to 6 months)	Astrocytes ↑↑↑ / microglia ↑↑↑ [16,19,20]
PLP1-rsh [51]	Spontaneous single amino acid substitution	yes [52]	Myelin ↓↓ / OL # ↔ / motor ↓↓ / normal lifespan	Astrocytes ↑↑ / microglia ↑↑ [18]
PLP1-jp [53]	Spontaneous deletion of exon 5		Myelin ↓↓↓ / OL # ↓↓↓ / motor ↓↓↓ / lethal (around P30)	Astrocytes ↑↑ / microglia ↑↑ [16,17]
PLP1-msd [54]	Spontaneous mutation of exon 6	yes [55]	Myelin ↓↓↓/ OL # ↓↓↓ / motor ↓↓ / lethal (around P30)	Astrocytes ↑↑↑ / microglia ↑↑↑ [15]

PLP mutant mice models are sorted by the severity of the pathophysiology, and summarized for type of PLP mutation, if mutation is found in clinical PMD population, major pathology (myelin/oligodendrocyte death/motor behavior ability/premature mortality) and reactive glia (astrocyte and microglia). Abbreviations: oligodendrocyte (OL); arrows indicate degree of change = no change ↔, mild ↑ or ↓, moderate ↑↑ or ↓↓, severe ↑↑↑ or ↓↓↓, or not reported (nr). Numbers in brackets represent relevant references.

Recently, two therapeutic approaches have been used in the PLP1tg mice that have indicated a possible involvement of gliosis and neuroinflammation in the PLP induced sequelae. Intriguing work from the Martini laboratory has provided strong evidence for microglia/macrophages and adaptive immunity (namely CD8^+^ T cells) in pathological changes in the PLP1tg mice [19,23,27-29]. By crossing the PLP1tg mice to a recombination activating gene-1 (RAG-1) mice, lacking mature T- and B-lymphocytes, they were able to reduce the number of CD11b cells and decrease the pathology in the PLP1tg mice [19]. A second approach involved feeding PLP1tg mice a diet enriched with cholesterol. The cholesterol enriched diet was found to rescue motor defects, but did not prevent the hypomyelination. It was suggested that the positive effects of a cholesterol-enriched diet in the PLP1tg mice could be mediated by maintaining health of oligodendrocytes and axons, as well as reducing gliosis and neuroinflammation [56].

Temporal and regional heterogeneity, which may be associated with the severity of the pathophysiology of different mutant PLP mice, has been reported. Interestingly in the PLP1^jp^ mice [53], which have missense mutation in the PLP1 gene, there is a progressive age-related increase in microglia that was restricted to white matter regions of the brain [16,17]. In the gray matter of the PLP1^jp^ mice, microglia density was increased compared to control mice, but there was no change with age [17]. However, the severe phenotype with short lifespan (around P30) in the PLP1^jp^ may mask more widespread and progressive microglia activation. For example in the PLP1-null mice, which have the mildest phenotype of the PLP mutant mice, microglia activation is found to be increased around one year of age. Interestingly, the increase in microglia activation corresponded with axonal degeneration but occurred before the motor impairment was evident [26]. While the regional distribution of microglia has not yet been reported in the PLP1dup mice, an increase in microglia number was found in regions of axon degeneration [21]. Moreover, microglia in the PLP1dup mice were found to have internalized degenerating axons [21].

Previously, we reported [35] that axonal degeneration was not seen in the optic nerve of the PLP-ISEdel mouse up to six months of age. However, in the current study we find markers of axonal injury (APP spheroids) in a number of brain regions in the CNS of the 4-month-old PLP-ISEdel mice. One potential explanation for the differences in these finding is the low level of microglia and astrocyte activation in the optic nerve. Previous works suggest that microglia are slow to respond to optic nerve injury [57]. However, in the PLPtg mice, CD11b^+^ cells were found to increase in number in the optic nerve at younger ages compared to other brain regions investigated [19]. APP accumulation represents a marker of disrupted anterograde axonal transport. In the PLP null mouse, APP^+^ swellings were identified in the optic nerve and cerebral white matter at approximately 2-months-old and were not found to be progressive [58]. In the current study we did not stain for APP or markers of glia activation in the optic nerve; therefore any comparison between our current study and previous work would be speculation. Interestingly, the regions of the brain with the most robust APP^+^ spheroids (thalamus and striatum) were also regions of the brain with strong microglia response and astrocyte reactivity. However, several mechanisms may contribute to the axonal degeneration. For example, the PLP-ISEdel mouse has impaired transport of sirtuin 2 from the oligodendrocyte cell body to the myelin membrane [59]. The absence of sirtuin 2, a deacetylase, in the myelin sheath may compromise signaling to the axons. In addition to cytokine secretion, activated microglia may contribute to defects of axonal integrity through sodium channel activation and excitoxicity, as has been shown in multiple sclerosis plaques (for review see [60]). The results presented here are descriptive observations; however, our findings do support the notion that regional heterogeneity in the reactive glia response may lead some areas of the CNS to be more susceptible to degeneration.

## Conclusions

Neuroinflammation has been suggested to be more damaging to axons than alterations in myelin integrity, including frank demyelination [58]. Our current finding of gliosis, along with other clinical reports of PMD cases [47], and preclinical models of PMD [16,19,23,24,26-29] highlight a potentially clinically relevant, oligodendrocyte independent response that may contribute to the disease progression. While more work is clearly necessary, neuroinflammation may represent a viable therapeutic target for PMD.

## Abbreviations

APP: Amyloid precursor protein; CNP: 2′,3′-cyclic-nucleotide 3′-phosphodiesterase; CNS: Central nervous system; GFAP: Glial fibrillary acidic protein; hip: hippocampus; IBA1: Ionized calcium-binding adapter molecule 1; IHC: Immunohistochemistry; ISE: Intronic splicing enhancer; KI: Knockin; KO: Knockout; Lamps: lysosomal associated mucin-like membrane proteins; MHCII: Major histocompatibility complex class II; PBS: Phosphate-buffered saline; PFA: Paraformaldehyde; LP: Proteolipid protein; PLP1: Proteolipid protein 1 gene; PMD: Pelizaeus-merzbacher disease; RAG-1: Recombination activating gene-1; SEM: Standard error of the mean; SPG2: Spastic paraplegia type 2; Cpu: Striatum (caudate putamen); TNF-α: Tumor necrosis factor-α; WT: Wild-type.

## Competing interests

The authors declare that they have no competing interests.

## Authors’ contributions

ADB, LVE, FC designed the research studies. ADB, SJW performed the experiments. ADB drafted the manuscript with the assistance of the other authors, who read and approved the final manuscript.
